# Assessment of Four Artificial Methods for Aging Plastic Mulch Films According to Efficiency, Rate, and Similarity to Natural Field-Aged Plastics

**DOI:** 10.1007/s10924-024-03481-5

**Published:** 2025-01-18

**Authors:** Martine Graf, Michaela K. Reay, Athanasios Dimitriou, David R. Chadwick, Davey L. Jones

**Affiliations:** 1 School of Environmental and Natural Sciences, Bangor University, Bangor, LL57 2UW UK; 2 Organic Geochemistry Unit, School of Chemistry, University of Bristol, Bristol, BS8 1TS UK; 3 The BioComposites Centre, Bangor University, Bangor, LL57 2UW UK

**Keywords:** ATR-FTIR, Polymer degradation, LDPE, PLA/PBAT, Plastic weathering, DSC/TGA

## Abstract

Artificial degradation is often used to recreate and accelerate the natural aging of plastic for small-scaled simulation experiments assessing their environmental impact. However, current artificial aging methods are rarely compared against reference materials or validated using field-aged samples, creating uncertainties when extrapolating results to naturally aged plastics, making it difficult to place findings in an environmentally applicable context. Therefore, here we compared four accessible, cost-effective, and easily replicable methods (heat, UVA, and UVC irradiance at two intensities) to produce artificially degraded materials. The artificial aging methods were assessed over a duration of 5 months against degradation rate, efficiency, and similarity to field-aged samples of conventional and biodegradable plastic mulch film over a 6-month field exposure period. We utilised attenuated total reflectance-Fourier transform infrared spectroscopy to calculate the carbonyl index and measure chemical changes of the mulch film surface, as well as differential scanning calorimetry and thermogravimetric analysis. Physical changes were assessed by thickness and surface roughness measurements. We found that UVA was the most suitable and realistic artificial degradation method at a medium rate, whilst UVC is recommended for rapid degradation without the need to simulate realistic changes, and heat for processing large volumes of samples without a requirement for realistic degradation over a prolonged time period. However, the methods compared in this study yielded differential results depending on polymer type and parameter of interest. We therefore recommend establishing the degradation aim, identifying the spectral region of interest, and accounting for different polymer types to select the most appropriate method.

**Supplementary Information:**

The online version contains supplementary material available at 10.1007/s10924-024-03481-5.

## Introduction

Environmental plastic pollution and its effect on ecosystem functioning has garnered increasing interest in recent decades. Understanding the effect of plastic on soil-plant interactions has become especially important in agroecosystems, where the continued application of agricultural plastic often leads to an accumulation of legacy plastic in arable soils [1]. One of the major contributors to macro- and microplastic pollution in fields is plastic mulch film, with an estimated global use of 2.5 million tonnes per year [1]. This has spurred the progressive adoption of degradable polymers (e.g., poly(lactic acid) (PLA), poly(butylene adipate-*co*-terephthalate) (PBAT), poly(3-hydroxybutyrate-*co*-3-hydroxyvalerate) (PHBV)) in plastic mulch film production to replace polyethylene-based mulch films [2]. The overall aim of this transition is to reduce legacy plastic in soil, as biodegradable mulch films are designed to degrade ≥ 90% within 24 months after application, in line with the EN 17033 EU standard [3]. However, it has already been shown that the degradation rate of these biodegradable films is highly influenced by environmental conditions, and often exceeds the targeted time period [4, 5].

To understand the effects of plastic mulch film residue on soil and plant properties, laboratory and pot experiments are often undertaken. Due to their small-scale nature, generally accelerated timeline, and the control of environmental variables compared to field experiments, they are ideal to investigate isolated effects or mechanisms driving change [6]. To date, this has greatly enhanced the overall understanding of plastic-soil-plant interactions and provided useful indications as to what requires closer examination in more realistic settings [7]. However, most studies utilise pristine plastic in a uniform size range and shape [8–10], which stands in contrast to the usual appearance of plastic residues in a natural soil environment [11, 12].

To create more realistic plastic material in a short timeframe, artificial degradation/aging methods are often used to alter the chemical structure and mechanical properties of polymers. These methods can be broadly divided into biotic (microbial and enzymatic) [13–15] and abiotic degradation, including chemical [16, 17], mechanical, UV radiation [18, 19], heat [20, 21], and other weathering processes [22]. Unfortunately, few studies using artificial degradation assess the degradation against reference materials or field-realistic samples, making it hard to quantify and qualify the achieved level of degradation in an environmentally applicable context. Whilst some standards for abiotic degradation are available [23] (e.g., ISO 4892-3 and ASTM D5208), they often require specialised testing equipment to simulate the appropriate environmental conditions [24], making their execution less accessible for a global research community.

Here, we therefore sought to evaluate four accessible, cost-effective, and easily reproducible methods to artificially degrade two common types of plastic mulch film and assess the chemical and physical changes against natural field weathering over 6 months in a temperate climate. The two polymers, low-density polyethylene (LDPE) and PLA/PBAT, were selected due to their common use in agricultural plastic mulch film production and subsequent relevance in agricultural plastic pollution [25]. The four degradation methods we compared were heat, UVA, and UVC irradiance at two intensities, reflecting increasing energy for abiotic degradation, and samples were exposed for a total of 20 weeks. These methods were selected due to their common use in current artificial degradation processes and production of reference materials in environmental research settings [18–20, 24, 26–28], as well as their accessibility and cost-effectiveness for a global research community. The chemical changes of the films were assessed weekly by attenuated total reflectance–Fourier transform infrared (ATR-FTIR) spectroscopy and quantified by calculation of the carbonyl index (CI). Physical changes to the mulch film surface were assessed after 20 weeks exposure, with focus on surface roughness and film thickness. We purposefully selected FTIR spectroscopy as the main analytical tool for this comparison study, as it is currently the most prevalent method used for polymer analysis in the field of environmental science [11, 12], making it an accessible and relatable method for a global research community with various capabilities and instrument access. The majority of research focusing on effects of plastic on the environment is utilising FTIR or similar spectroscopy methods for polymer identification and degradation assessments [29, 30], making it therefore the most relevant method for the analysis of artificially degraded reference materials. However, we also utilised differential scanning calorimetry (DSC) and thermogravimetric analysis (TGA) as complimentary analytical tools to assess polymer degradation for the endpoints of all exposure methods.

The three main objectives of this study were to evaluate the four methods according to (i) their degradation efficiency; (ii) their degradation rate and overall required timeframe for degradation to occur; and (iii) their similarity to natural field exposure.

## Materials and Methods

### Plastic Mulch Film Material

The two mulch film types used were a conventional LDPE (Gro-Clean, GroMax, Suffolk, UK; colour: black; thickness: 23 μm) and a biodegradable PLA/PBAT (ratio 85% PBAT; 15% PLA with a polybutylene sebacate adhesive [31]; Gro-Clean Bio-Mulch, GroMax, Suffolk, UK; colour: black; thickness: 10.5 μm) film. Organic co-contaminants were identified using a sequential solvent extraction followed by gas chromatography (GC) and GC/MS (mass spectrometry) analyses, and fillers and metals were identified by analysing the ash content of the mulch films using total reflection X-rays fluorescence (Reay et al., under review). Both mulch film types showed a high filler and metal content compared to their corresponding additive content. The most abundant additive groups for LDPE were lubricants > antioxidants > plasticisers, alongside non-intentionally added substances (e.g. ethoxylated amines as degradation products of antistatic agents). For PLA/PBAT, the most abundant additives were plasticisers > lubricants > chain extenders > antioxidants. Similar to LDPE, there were non-intentionally added substances, dominated by cyclic oligomers derived from the polymer itself [31], and degradation of additives beyond the analytical window (e.g. pentaerythritol fatty acid esters).

### Field and Artificial Exposure Methods

The study site for realistic field degradation was an arable field located at the Henfaes Research Centre, Abergwyngregyn, North Wales, UK (53°14’20’’N, 4°00’55’’W). The site has a temperate oceanic climate, and environmental data regarding air temperature, precipitation, solar radiation, wind speed, and relative humidity were recorded in 30-minute intervals by a meteorological station on site. Over the 6-month period, the average air temperature was 15.2 °C, total precipitation was 502 mm, average relative humidity was 78.5%, average solar irradiance was 132.7 W m^− 2^, and average wind speed was 10.7 km h^− 1^. Monthly average values for each sampling time point can be found in Table S1.

The plastic mulch films were laid onto rotavated 4 m x 1.2 m plots and secured by burying the edges at 10 cm soil depth. Plots were arranged in a 4-block design with random plot allocation for each mulch film type within each block, with 4 replicates per mulch film. *Zea mays* L. was grown to harvest through the two plastic mulch films with a seed density of 14 seeds m^− 2^ to simulate realistic mulch film use in an agricultural setting (Fig. S1). A 10 cm x 10 cm exposed film sample was taken in monthly intervals from each plot, randomising sampling location within each plot. Mulch films were first laid out onto the field on 31st May 2022 and removed on 28th November 2022.

For the artificial degradation, four laboratory methods were compared, and new, unexposed film samples were exposed for a duration of 20 weeks with weekly sampling intervals. The first method was heat exposure (oven-aging), using an 80 °C standard fan-oven (FED 400; Binder GmbH, Germany) [28, 32]. LDPE and PLA/PBAT films were cut into 5 cm x 5 cm pieces (*n* = 100), placed into two separate glass beakers, and left in the oven. Film pieces in each beaker were mixed at each sampling point to prevent adhesion, and samples for analysis (*n* = 4) were selected randomly from each beaker and removed.

The second method was UVA irradiance [27, 30, 33] using a Suntest XXL + FD light stability test chamber (Atlas Material Testing Technology, Illinois, USA) with 3 × 1700 W xenon lamps with wavelength set to 300–400 nm and irradiance at 60 W m^− 2^, a chamber temperature of 25 °C, and relative humidity of maximum 50%. Due to the constant airflow in the chamber, individual film pieces could not be secured, therefore a 35 cm x 35 cm piece of each mulch film was placed into the test chamber and secured at the corners, with an average 40 cm distance from the light bulbs. At each sampling point, the mulch films were removed for spectral analysis, and afterwards a 5 cm x 5 cm piece of each film was removed for storage (*n* = 1) before placing the films back into the chamber. After an exposure time of 20 weeks, this equates to a UVA dose of 725.8 MJ m^− 2^. This method is more representative of natural weathering and involves deeper penetration of the UVA (in comparison to UVC) which might lead to bulk property changes.

The third method was low UVC irradiance [26, 34] at an average of 1.6 W m^− 2^ (measured at wavelength range of 220–275 nm) using a LabGard Class II biosafety cabinet (NuAire Inc., Minnesota, USA) with a standard 30 W UVC lamp. Due to small chamber space, mulch films were cut into 5 cm x 5 cm pieces (*n* = 4) and placed centrally under the light bulb at 68 cm distance from the light source. Samples were removed at each sampling point for spectral analysis, then returned to the UVC cabinet with rotating positions to ensure even UV exposure of all samples. After an exposure time of 20 weeks, this equates to a UVC dose of 19.4 MJ m^− 2^. The higher energy of UVC is expected to cause more immediate surface interactions, leading to more surface-concentrated degradation, less penetration depth, and more rapid surface modifications.

Lastly, the fourth method was high UVC irradiance at an average of 3.9 W m^− 2^ (measured at wavelength range of 220–275 nm) using the same exposure method as described for low UVC irradiance, but with a 16 cm distance from the light source. After an exposure time of 20 weeks this equates to a UVC dose of 47.2 MJ m^− 2^.

The exposure methods are from this point onwards referred to as field, heat, Suntest, low UVC, and high UVC exposure, respectively. Control spectra were taken from new, unexposed films, from here on referred to as control.

### Analysis of Chemical Polymer Changes

To assess changes in the chemical spectra of the mulch films at each time point, ATR-FTIR spectroscopy was performed. The analysis was carried out using a Cary 630 FTIR bench spectrometer (Agilent Technologies Inc., California, USA) with a diamond ATR crystal attachment in conjunction with a KBr source with a spectral range of 650–4000 cm^− 1^ and a resolution of 4 cm^− 1^. Spectra were recorded as reflectance % (32 scans per sampling point & background) using the MicroLab Pharma Software (Agilent Technologies Inc., California, USA). The crystal was routinely cleaned using acetone, and background measurements were taken every 2 min. Five random points per sample (*n* = 4) were measured, and sides (upper and lower) were randomly switched throughout the sampling process, resulting in a total of 20 spectra per film type per time point. For the Suntest samples, 20 sampling points were recorded for the large film sample at each time point, using the same sampling approach.

Spectra manipulation and transformation were carried out using the open source online application Open Specy [35]. Spectra were transformed to absorbance according to the Kubelka-Munk equation (where the absorbance coefficient is calculated using the measured reflectance (*R*): (1-*R*)^2^/2*R), baseline correction was carried out with the setting ‘Polynomial 8’, and the CO_2_ region between 2200 and 2400 cm^− 1^ was flattened. Processed spectra were then downloaded and used for further analysis.

To quantify changes in peak intensity, the area under the carbonyl (C=O) peak and a reference peak (here: mix of carbon-hydrogen (CH) bonds) was calculated and the carbonyl index (CI) determined by dividing the area under the C=O peak by the area under the CH peak [36]. The CI is used as a quantification tool for the carbonyl peak evolution and oxidation of the polymers during photo or thermal oxidation [36], leading to chain scission of CH and C-C bonds and formation of carbonyl groups, present in aldehydes, ketones and carboxylic acids, in the presence of oxygen. To capture the entire peak area and account for peak shifts with prolonged exposure, the C=O wavenumber interval of 1504–1976 cm^− 1^ [37, 38] and 1560–1808 cm^− 1^ [39–42], and CH wavenumber interval of 1384–1480 cm^− 1^ [37, 43, 44] and 1304–1504 cm^− 1^ [45–50] were used for LDPE and PLA/PBAT mulch film, respectively. For LDPE, the C=O wavenumber interval includes C=O stretching vibrations, and bending vibrations of CH bonds, and the CH wavenumber interval includes bending or scissoring vibrations of CH bonds (primarily CH_2_) as well as some CH stretching. For PLA/PBAT, the C=O wavenumber interval includes stretching vibrations of the C=O group, C-C stretching or bending vibrations, and bending vibrations of CH bonds, and the CH wavenumber interval includes the bending or scissoring of CH bonds (primarily CH_3_ and CH_2_).

### Analysis of Thermal Polymer Changes

To compliment the ATR-FTIR analysis, which provides information on surface changes to the polymer, but may be influenced by variation in sample thickness and penetration depth, therefore not entirely representative of the bulk sample, differential scanning calorimetry (DSC) and thermogravimetric analysis (TGA) were carried out on the end points of all samples, as well as the controls (*n* = 3). The DSC and TGA analyses were carried out using a TGA/DSC 1 STARe System (Mettler Toledo, Ohio, USA). Samples were weighed out to 5 mg ± 1 mg into 70 µl aluminium oxide crucibles (Mettler Toledo, Ohio, USA), and analysed using the following settings under 100 ml min^− 1^ N_2_ flow: (i) heat cycle 1: 25–210 °C at a rate of 10 °C min^− 1^; (ii) hold at 210 °C for 3 min; (iii) cooling cycle: 210–25 °C at a rate of 10 °C min^− 1^; (iv) heat cycle 2: 25–800 °C at a rate of 10 °C min^− 1^. The first heat cycle was used to eliminate any thermal history of the polymers, and the second heat cycle was used for the DSC and TGA analyses.

### Analysis of Physical Polymer Changes

Physical polymer changes were measured on control samples, all field exposure time points, and the final 20 weeks exposure time points of the artificial degradation methods. Film thickness [µm] was measured using a thickness gauge (Mitutoyo, Kawasaki, Japan) and surface roughness (*Ra*) [µm] was measured using a surface roughness tester (Surfest SJ-210; Mitutoyo, Kawasaki, Japan) following the ISO 4287:1997 standard with speed set to 0.5 mm s^− 1^, total measured length = 12.5 mm, *λ* = 2.5, and measurements split into 5 equal parts to calculate *Ra*. For both measurements, each sample (*n* = 4) was measured 5 times and sides were randomly switched throughout the sampling process, resulting in a total of 20 measurements per time point.

### Statistical Analysis

Statistical analysis and data visualisation were carried out using R version 4.3.3 [51]. Non-metric multi-dimensional scaling (NMDS) plots based on Bray-Curtis distance were created using packages ‘vegan’ version 2.6-4 [52] and ‘ggplot2’ version 3.5.0 [53]. Spectra and DSC/TGA graphs were created using package ‘ggplot2’. Graphs for CI and changes in peak area were created using Microsoft Excel. All graphs were edited using Affinity Designer version 1.10.5.1342 (Serif Ltd., UK). Differences between exposure time for film thickness, surface roughness, and peak area changes were determined by One-way ANOVA with subsequent Tukey HSD test with a 95% confidence interval (*p* ≤ 0.05).

## Results

### Chemical Polymer Degradation

**Fig. 1:**
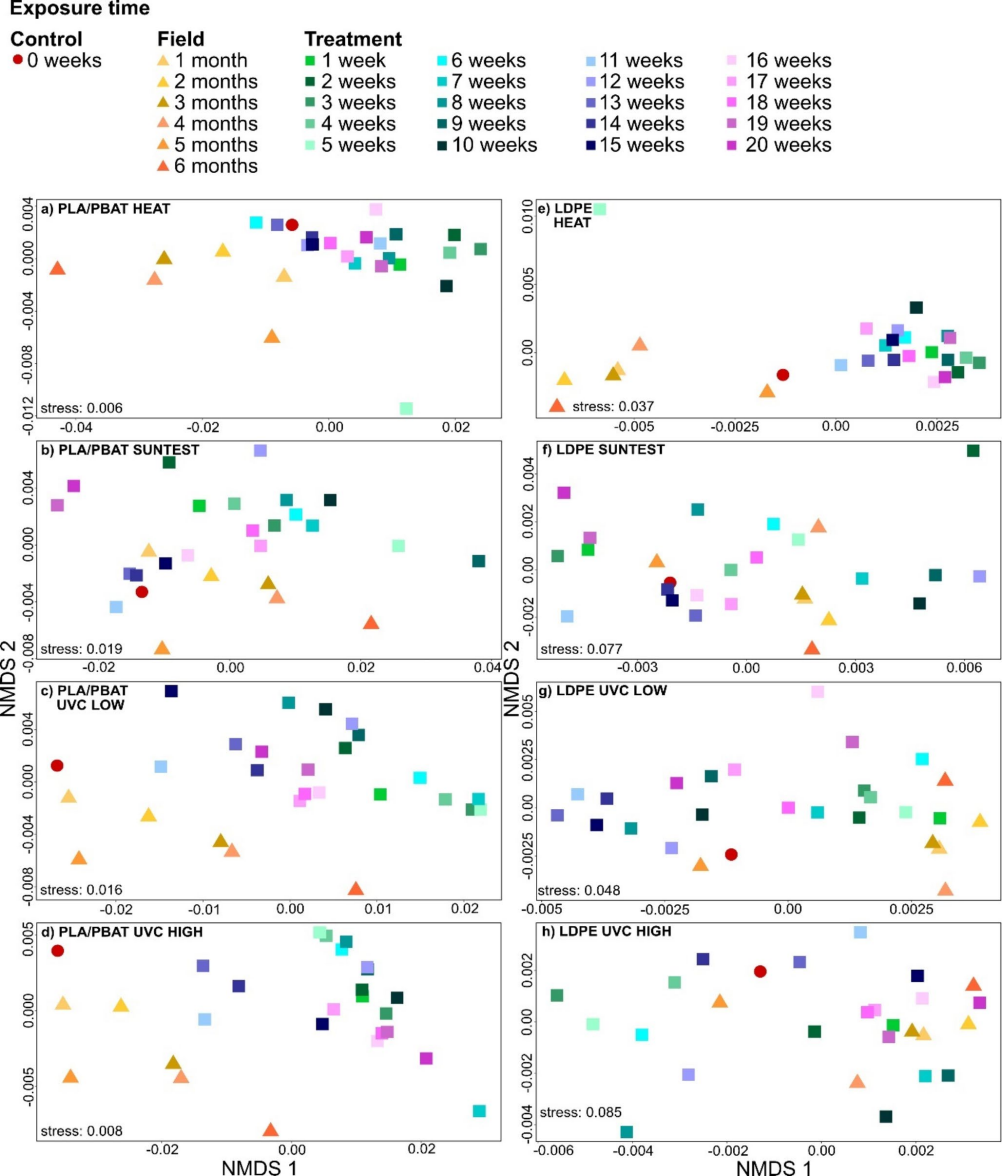
Non-metric multi-dimensional scaling (NMDS) plots based on Bray-Curtis distance showing the data distribution of control (0 weeks), field (1–6 months), and treatment (1–20 weeks) exposure time. Data points are expressed as mean (*n* = 20). **(a)** PLA/PBAT mulch film control, field, and heat exposure. **(b)** PLA/PBAT mulch film control, field, and Suntest (UVA) exposure. **(c)** PLA/PBAT mulch film control, field, and UVC exposure at low intensity. **(d)** PLA/PBAT mulch film control, field, and UVC exposure at high intensity. **(e)** LDPE mulch film control, field, and heat exposure. **(f)** LDPE mulch film control, field, and Suntest (UVA) exposure. **(g)** LDPE mulch film control, field, and UVC exposure at low intensity. **(h)** LDPE mulch film control, field, and UVC exposure at high intensity

Overall similarities between control, field, and artificial degradation methods for all time points are visualised in NMDS plots (Fig. 1) with all stress values indicating a fair to good fit. Stress values indicate how well the displayed 2-dimensional distance between sample points corresponds with the real multivariate distance between samples. Lower stress values indicate good conformity, whereas higher stress values indicate an arbitrary sample distribution. The Bray-Curtis distance was used to quantify how different/similar spectra are based on their relative intensity values across wavenumbers. The plots display the mean (*n* = 20) value of all exposure time points, with similar samples being grouped closer together and dissimilar samples being farther apart, i.e. the more similar the average intensity values of the spectra of samples are to each other, the closer they are in the NMDS plot, and vice versa.

**Fig. 2:**
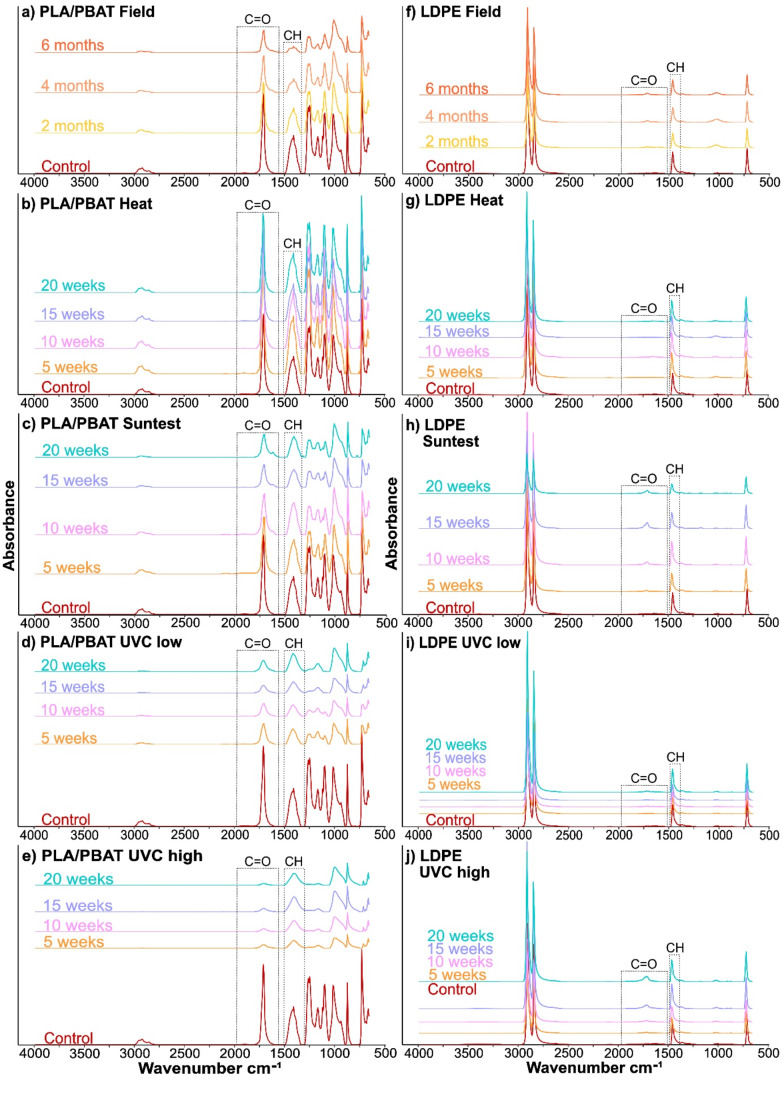
ATR-FTIR absorbance spectra in the range of 650–4000 cm^− 1^. Spectra are expressed as mean (*n* = 20). Graphs show a selection of time points for **(a)** PLA/PBAT mulch film field exposure; **(b)** PLA/PBAT mulch film heat exposure; **(c)** PLA/PBAT mulch film Suntest (UVA) exposure; **(d)** PLA/PBAT mulch film UVC exposure at low intensity; **(e)** PLA/PBAT mulch film UVC exposure at high intensity; **(f)** LDPE mulch film field exposure; **(g)** LDPE mulch film heat exposure; **(h)** LDPE mulch film Suntest (UVA) exposure; **(i)** LDPE mulch film UVC exposure at low intensity; and **(j)** LDPE mulch film UVC exposure at high intensity. Dotted lines show carbonyl (C=O) and carbon-hydrogen (CH) peak areas used for carbonyl index calculations

**Fig. 3:**
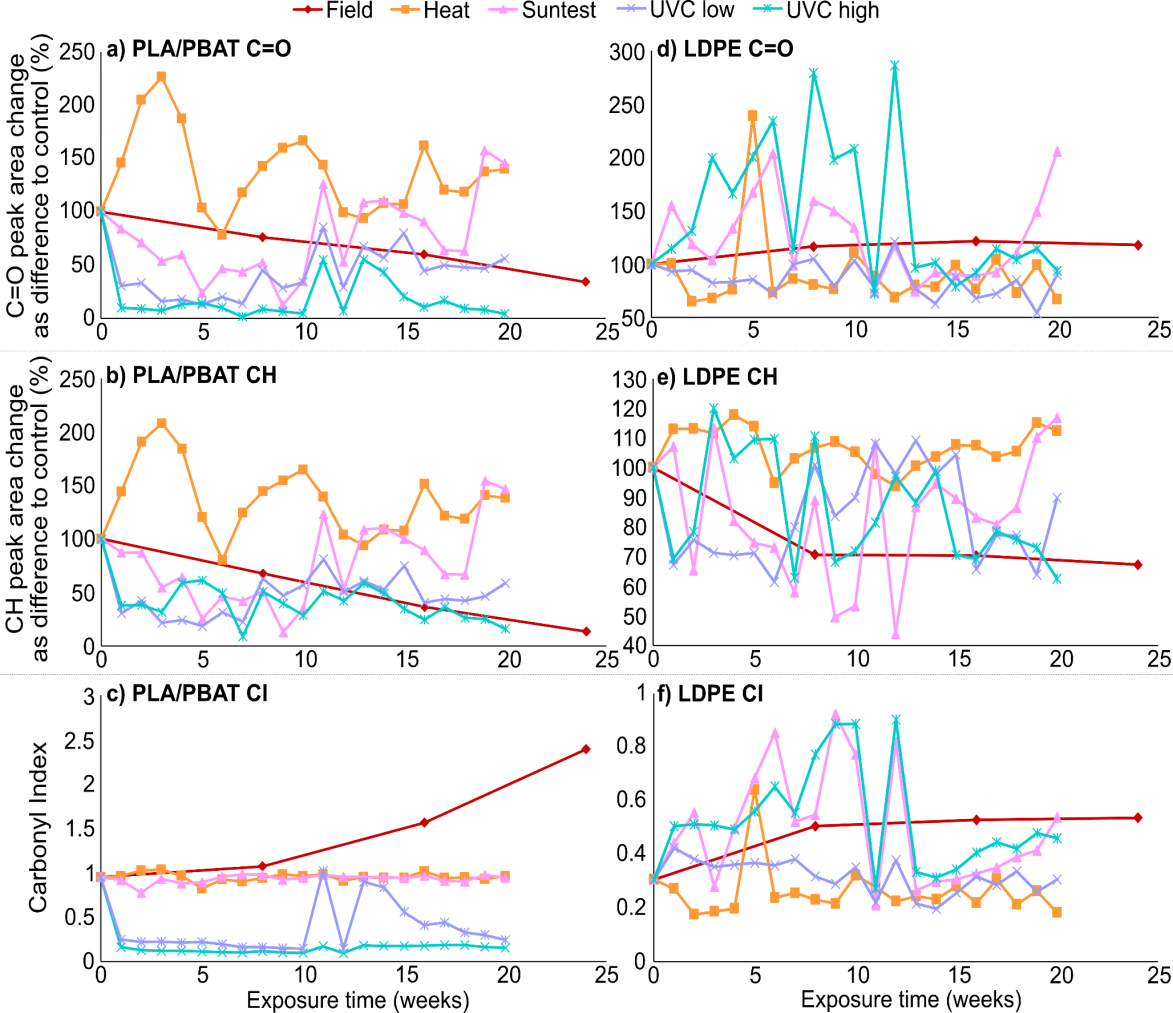
Peak area change relative to the control and carbonyl index for absorbance spectra of PLA/PBAT and LDPE plastic mulch film exposed to different weathering conditions. Values expressed as mean (*n* = 20). **(a)** PLA/PBAT C=O peak area change expressed as difference to control in wavenumber interval 1560–1808 cm^− 1^; **(b)** PLA/PBAT CH peak area change expressed as difference to control in wavenumber interval 1304–1504 cm^− 1^; **(c)** PLA/PBAT carbonyl index expressed as C=O peak area / CH peak area; **(d)** LDPE C=O peak area change expressed as difference to control in wavenumber interval 1504–1976 cm^− 1^; **(e)** LDPE CH peak area change expressed as difference to control in wavenumber interval 1384–1480 cm^− 1^; **(f)** LDPE carbonyl index expressed as C=O peak area / CH peak area

Changes in peak intensity and area are shown as mean values (*n* = 20) for a selection of exposure time points to showcase gradual changes over time against the control spectra (Fig. 2, S2, S3). The resulting peak area changes of the C=O and CH regions, as well as the CI are displayed as difference to the control spectra (Fig. 3).

#### PLA/PBAT Spectra Changes after Exposure

The distance between control and field exposure samples increased progressively with increasing exposure time, indicating a gradual spectral change over time (Fig. 1a-d). This was also showcased in the overall gradual reduction of peak intensities (Fig. 2a, S2a, f) and steady decline of the C=O and CH peak areas, with a 65% (*p* < 0.001) and 87% (*p* < 0.001) reduction compared to control after 6 months, respectively (Fig. 3a-b), resulting in a CI increase of 1.4 after 6 months (Fig. 3c).

Heat exposure samples were clustered in the opposite direction to the field exposure samples, with distance increasing with increasing field exposure time (Fig. 1a). Further, the spectra changes confirmed that only slight changes in peak intensities occurred with increasing exposure time, overall remaining similar to the control (Fig. 2b, S2b, g). Both C=O and CH peak areas showed high variation, but followed a similar trend in terms of difference to control with prolonged exposure (Fig. 3a-b), with a maximum increase of 126% and 107% after 3 weeks, and a 40% and 38% increase after 20 weeks for C=O and CH peak areas, respectively, resulting in a relatively stable CI with an increase of only 0.02 after 20 weeks exposure (Fig. 3c). Overall, early heat exposure samples (5 and 10 weeks) showed a significant difference to control for C=O and CH peak area whereas prolonged exposure showed no difference (Table S2, Fig. S2b, g).

Suntest (UVA) exposure samples followed a similar trend to early field exposure samples but became more distant with increasing field exposure time (Fig. 1b). Changes in the spectra showed a more rounded CH peak with increased exposure compared to the field samples, and an additional small shoulder peak in the C=O region at 1621 cm^− 1^, which was also present in field samples, though less pronounced (Fig. 2a, c, S2c, h). The C=O and CH peak area changes of the Suntest exposure samples showed high variation but followed a similar trend, resulting in a relatively stable CI similar to the heat exposure sample CI (Fig. 3a-c), with a decrease of only 0.01 after 20 weeks compared to the control. Both areas showed an initial rapid decline with short exposure time, with a maximum decline of 88% and 87% at 9 weeks and an increase of 45% and 46% at 20 weeks, for C=O and CH, respectively. Early Suntest exposure samples (5 and 10 weeks) showed no difference to the control for C=O and CH peak area, whereas prolonged exposure showed a significant difference to control spectra (Table S2).

Low UVC irradiated samples displayed a similar directional data distribution to field samples (Fig. 1c), with spectra showing a more rapid reduction and smoothing of peaks with increased exposure time (Fig. 2d, S2d, i). Both C=O and CH peak areas showed an initial rapid decline with a slight increase with prolonged exposure time at around 11 weeks (Fig. 3a-b), leading to a low CI with increased variation for later weeks (Fig. 3c). The peak areas decreased at a maximum of 87% and 82% at 5 weeks, with a lesser decrease of 44% and 41% at 20 weeks for C=O and CH, respectively, with both peak areas differing significantly from the control spectra (Table S2). The CI showed an immediate decline and remained relatively stable up to 10 weeks (decline of 0.85), then showed more variation and settled at a decrease of 0.79 at 20 weeks.

High UVC irradiated samples showed a similar trend to low UVC samples in terms of spatial distribution to field samples (Fig. 1d), but spectra showed a more accelerated peak intensity decline and smoothing (Fig. 2e, S2e, j) with a slightly faster reduction in the C=O area compared to CH (Fig. 3a-b). The maximum peak area decreased by 98% and 91% at 7 weeks exposure, with a final decrease of 96% and 84% at 20 weeks, for C=O and CH peak areas, respectively, with both peak areas differing significantly from the control spectra (Table S2). The CI decreased rapidly after exposure start, with a maximum decrease of 0.8 at 10 weeks and a final decrease of 0.7 at 20 weeks (Fig. 3c).

#### PLA/PBAT Functional Group Peak Area Comparison to Field Exposure

The comparison of the C=O peak area of selected artificial exposure time points to control and selected field exposure time points showed that heat 15 (*p* = 0.99) and 20 weeks (*p* = 0.99), and Suntest 5 (*p* = 0.99) and 10 weeks (*p* = 0.91) were similar to the control spectra. Whereas field exposure samples after 2 months showed the highest similarity to heat 15 weeks (*p* = 0.36), heat 20 weeks (*p* = 0.16), and Suntest 5, 10, 15, and 20 weeks (*p* = 0.61; 0.99; 0.13; 0.86, respectively). The most similar exposure methods and time points for 4 months field exposure were Suntest 15 (*p* = 0.99) and 20 weeks (*p* = 0.99), and low UVC 5 (*p* = 0.99) and 10 weeks (*p* = 0.89). For 6 months field exposure, the most similar exposure points were Suntest 15 (*p* = 0.99) and 20 weeks (*p* = 0.58), and low UVC at 5, 10, 15, and 20 weeks (*p* = 0.99; 0.99; 0.82; 0.99, respectively).

Likewise, the CH peak area for control samples was most similar to heat 15 (*p* = 0.99) and 20 weeks (*p* = 0.99), and Suntest 5 (*p* = 0.99) and 10 weeks (*p* = 0.98). Field exposed samples after 2 months showed the highest similarity to Suntest 10 (*p* = 0.21), 15 (*p* = 0.69), and 20 weeks (*p* = 0.99), low UVC 5 (*p* = 0.25) and 20 weeks (*p* = 0.99), and high UVC 15 (*p* = 0.91) and 20 weeks (*p* = 0.21) exposure. For 4 months field exposure, the most similar artificial exposure time points were Suntest 15 (*p* = 0.97) and 20 weeks (*p* = 0.33), low UVC exposure at 5, 10, 15, and 20 weeks (*p* = 0.99; 0.99; 0.99; 0.48, respectively), and high UVC exposure at 5, 10, 15, and 20 weeks (*p* = 0.99; 0.99; 0.85; 0.99, respectively). Lastly, 6 months field samples were closest to low UVC exposure at 10 (*p* = 0.68) and 15 weeks (*p* = 0.52), and high UVC at 5 (*p* = 0.99) and 10 weeks (*p* = 0.19). All other exposure time points were either significantly different, or close to significance, to control and field exposure (Table S2).

#### LDPE Spectra Changes after Exposure

Field exposed sample points were overall clustered and distanced from the control, with exception of 5 months presenting an outlier with closer proximity to the control (Fig. 1e-h). The spectra showed a gradual increase in C=O peak intensity with prolonged exposure, and a slight decrease in the CH peak intensity (Fig. 2f, S3a, f). This was also reflected in the relatively stable increase of the C=O area of 17% (*p* = 0.99) after 6 months (Fig. 3d) and decline of the CH area of 37% (*p* < 0.001) (Fig. 3e) compared to the control, leading to an initial increase and later stabilisation of the CI, with an increase of 0.23 after 6 months exposure (Fig. 3f). The field exposure of PLA/PBAT led to more significant changes in the CI likely due to the additional environmental variables affecting the degradation of the mulch film in the field. PLA/PBAT is biodegradable, therefore combined exposure to UV, mechanical stress from wind and plants fracturing the surface, precipitation, and direct contact with the soil surface allowing for microbial degradation, all affected the degradation dynamics. Although LDPE mulch film was exposed to the same conditions, it is not biodegradable, therefore the effect of the additional environmental degradation was less pronounced.

Heat sample points showed a clear spatial separation from control and field exposure samples (Fig. 1e), and spectra showed no obvious difference in peak intensity compared to the control (Fig. 2g, S3b, g). However, a closer look at the peak area changes revealed a maximum decrease of the C=O area of 35% at 2 weeks and 33%, and an increase of the CH area of 17% at 4 weeks and 12% at 20 weeks, with both peak areas remaining relatively stable with fluctuations throughout and no significant difference to the control spectra (Table S3). This was reflected in a relatively stable CI, with exception of week 5, with a decrease of 0.12 after 20 weeks.

The Suntest data points showed a relatively homogenous distribution with the field exposure samples (Fig. 1f), and a similar increase and decrease in C=O and CH peak intensity, respectively, compared to the field samples (Fig. 2h, S3c, h). The peak area changes compared to the control were highly variable, with an increase of 105% at 20 weeks for C=O, and a maximum decrease of 56% at 12 weeks with a final decrease of 17% at 20 weeks for CH (Fig. 3d-e). Significant differences to the control in the C=O peak area appeared at 15 weeks (*p* < 0.001), and at 15 (*p* = 0.007) and 20 (*p* < 0.001) weeks for CH, whilst other time points remained similar to the control (Table S3). This resulted in an extremely variable CI with an initial increase, followed by a rapid decrease at around 13 weeks, and a final slow increase, with a maximum increase of 0.62 after 9 weeks and 0.23 increase at 20 weeks compared to control (Fig. 3f).

The initial low UVC data points were distributed similarly to the field samples but diverged in the opposite direction with increasing exposure time (Fig. 1g). Overall, the spectra showed only slight changes to the peak intensities compared to the control (Fig. 2i, S3d, i) with the C=O peak area slightly decreasing by 10% after 20 weeks and remaining relatively stable throughout (Fig. 3d) and similar to the control for all time points (Table S3). On the other hand, there was more variation in the CH peak area, with a maximum decrease of 30% at 4 weeks and a final decrease of 10% at 20 weeks (Fig. 3e), leading to a significant difference to the control CH peak area for 5, 10, and 15 weeks exposure (*p* < 0.001 for all). The CI remained stable throughout, with a maximum decrease of 0.11 at 14 weeks and no change to the control at 20 weeks (Fig. 3f).

In contrast to the low UVC, high UVC data points with prolonged exposure were clustered closer to field exposure points, whereas early high UVC exposure points were farther removed (Fig. 1h). The spectra showed an increase of the C=O peak intensity with increasing exposure time and only slight changes for the CH peak intensity (Fig. 2j, S3e, j). The peak area changes for C=O and CH were extremely variable, and showed a maximum increase of 186% at 12 weeks and a 6% decrease at 20 weeks for C=O, and maximum increase of 20% at 3 weeks and decrease of 38% at 20 weeks exposure for CH. The C=O peak areas of 15 (*p* < 0.001) and 20 weeks (*p* < 0.001) and the CH peak areas of 5 (*p* < 0.001) and 10 weeks (*p* < 0.001) exposure differed significantly from the control, with remaining time points showing no significant difference (Table S3). Similar to the Suntest CI, the high UVC CI increased rapidly (increase of 0.6 at 12 weeks) before declining sharply at 13 weeks, to gradually increase again up to 20 weeks with an increase of 0.15.

#### LDPE Functional Group Peak Area Comparison to Field Exposure

The comparison of the C=O peak area of selected artificial exposure time points to control and field exposure at 2, 4, and 6 months showed no significant differences, except for Suntest 15 weeks (*p* < 0.001 for all), and high UVC 15 and 20 weeks (*p* < 0.001, respectively for both).

The CH peak area comparison on the other hand was more variable, and showed that the control was most similar to heat 5, 10, 15, and 20 weeks (*p* = 0.99; 0.85; 0.99; 0.99, respectively), Suntest 5 and 10 weeks (*p* = 0.34; 0.99, respectively), low UVC 20 weeks (*p* = 0.99), and high UVC samples at 15 and 20 weeks exposure (*p* = 0.69; 0.99, respectively). Samples after 2 months field exposure were most similar to Suntest 5 and 15 weeks (*p* = 0.77; 0.99, respectively), low UVC samples at 5, 10, and 15 weeks (*p* = 0.99 for all), and early high UVC samples at 5 and 10 weeks exposure (*p* = 0.99 for both). For 4 months field exposure, the most similar artificial exposure time points were Suntest 5 and 15 weeks (*p* = 0.71; 0.99, respectively), low UVC at 5, 10 and 15 weeks (*p* = 0.99 for all), and early high UVC at 5 and 10 weeks exposure (*p* = 0.99 for both). Lastly, the CH area of 6 months field exposure was most similar to Suntest 5 and 15 weeks (*p* = 0.32; 0.99, respectively), low UVC at 5, 10, and 15 weeks (*p* = 0.99 for all), and early high UVC at 5 and 10 weeks exposure (*p* = 0.99 for both).

All other exposure time points were either significantly different, or close to significance, to control and field exposure (Table S3).

### Thermal Polymer Changes

#### PLA/PBAT

**Fig. 4:**
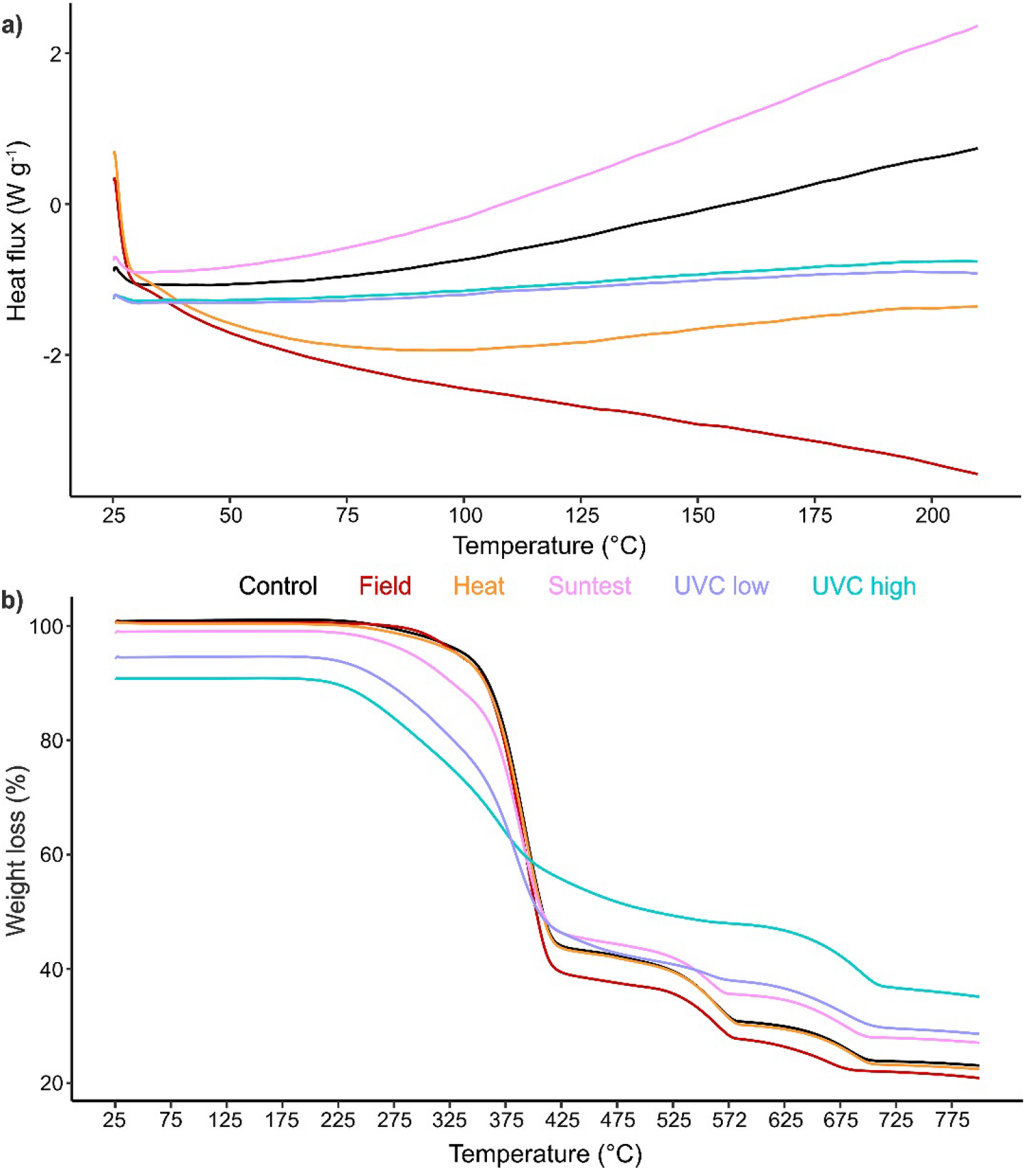
DSC **(a)** and TGA **(b)** curves of PLA/PBAT for control, field exposed samples at 6 months, and heat, Suntest (UVA), low UVC, and high UVC exposure at 20 weeks. Values expressed as mean (*n* = 3)

The comparison of the thermal properties of control, field exposed samples at 6 months, and artificially degraded samples at 20 weeks using DSC, showed no clearly defined melting temperature (*T*_*m*_) for any of the samples (Fig. 4a). This could be due to an overall reduced crystallinity in the polymer blend, which was dominated by PBAT, potentially masking the PLA signal. The crystallinity of the polymer was likely impacted by the production process, resulting in an amorphous blend, to make the mulch film more flexible for its intended use.

The residual sample weight at 800 °C (*m*_*800*_) during the TGA was relatively high for all samples (> 20%) and increased for Suntest and both UVC exposure methods compared to the control, whereas heat and field exposed samples remained similar to the control (Table 1). The high weight residue is likely attributed to char formation from PBAT and residue of fillers and degradation by-products that did not fully decompose. The increase in *m*_*800*_ under intense UV irradiance (Suntest and both UVC exposure methods) could be due to the formation of stable residues from crosslinking and oxidation during the degradation process, or a prior shift in the polymer:filler ratio, resulting in a higher residual mass %.

**Table 1:** Remaining mass, onset decomposition temperature(s), and melting temperatures after TGA and DSC analyses for PLA/PBAT and LDPE. Sampling timepoints tested were control, field exposure after 6 months, and artificial exposure methods after 20 weeks

	LDPE				PLA/PBAT			
	*m*_*800*_ *[%]*	*T*_*d*_ *[°C]*	*T*_*m1*_ *[°C]*	*T*_*m2*_ *[°C]*	*m*_*800*_ *[%]*	*T*_*d1*_ *[°C]*	*T*_*d2*_ *[°C]*	*T*_*d3*_ *[°C]*
Control	2.59 ± 1.21	435.42 ± 1.56	106.33 ± 1.42	122.22 ± 0.22	23.09 ± 1.57	343.39 ± 0.56	535.94 ± 0.78	650.22 ± 1.48
Field	6.90 ± 2.75	434.03 ± 1.55	109.89 ± 0.06	122.66 ± 0.38	20.90 ± 0.97	323.27 ± 1.26	533.61 ± 2.25	642.99 ± 6.5
Heat	5.75 ± 0.67	434.43 ± 0.47	104.17 ± 0.29	122.16 ± 0.19	22.48 ± 0.89	344.86 ± 0.55	531.61 ± 1.5	659.16 ± 2.64
Suntest	6.01 ± 0.76	429.83 ± 1.42	102.33 ± 1.55	121.50 ± 0.16	27.07 ± 2.25	260.27 ± 1.71	528.5 ± 0.33	644.15 ± 2.3
UVC low	3.87 ± 1.01	434.77 ± 0.39	110 ± 0	122.56 ± 0.34	28.61 ± 2.80	230.83 ± 2.02	532.83 ± 4.7	646.5 ± 3.35
UVC high	3.66 ± 0.54	428.78 ± 1.16	106.61 ± 1.23	121.61 ± 0.89	35.12 ± 1.02	224.83 ± 2.3	n/a	652.83 ± 2.26

Values expressed as mean ± SE (*n* = 3). *m*_*800*_ = remaining mass at 800 °C after TGA; *T*_*d1−3*_ = onset decomposition temperature(s); *T*_*m1−2*_ = melting temperature(s) during DSC

Similarly, the TGA curves showed drastic changes from the control for Suntest and both UVC exposure methods, with heat and field exposure methods aligning more closely with the control graph (Fig. 4b). The curves showed three separate degradation onset temperatures (*T*_*d1−3*_) and an overall reduction in *T*_*d*_ after degradation occurred, with the highest reduction observed for high UVC exposed samples, where no *T*_*d2*_ was identifiable (Fig. 4b; Table 1). The presence of multiple steps in the thermal decomposition can likely be attributed to the distinct decomposition temperatures of PLA and PBAT, their interaction with each other, and the presence of polybutylene sebacate adhesive and additives. The overall decrease in *T*_*d1−3*_ after degradation indicates that the polymer blend has become less thermally stable.

#### LDPE

**Fig. 5:**
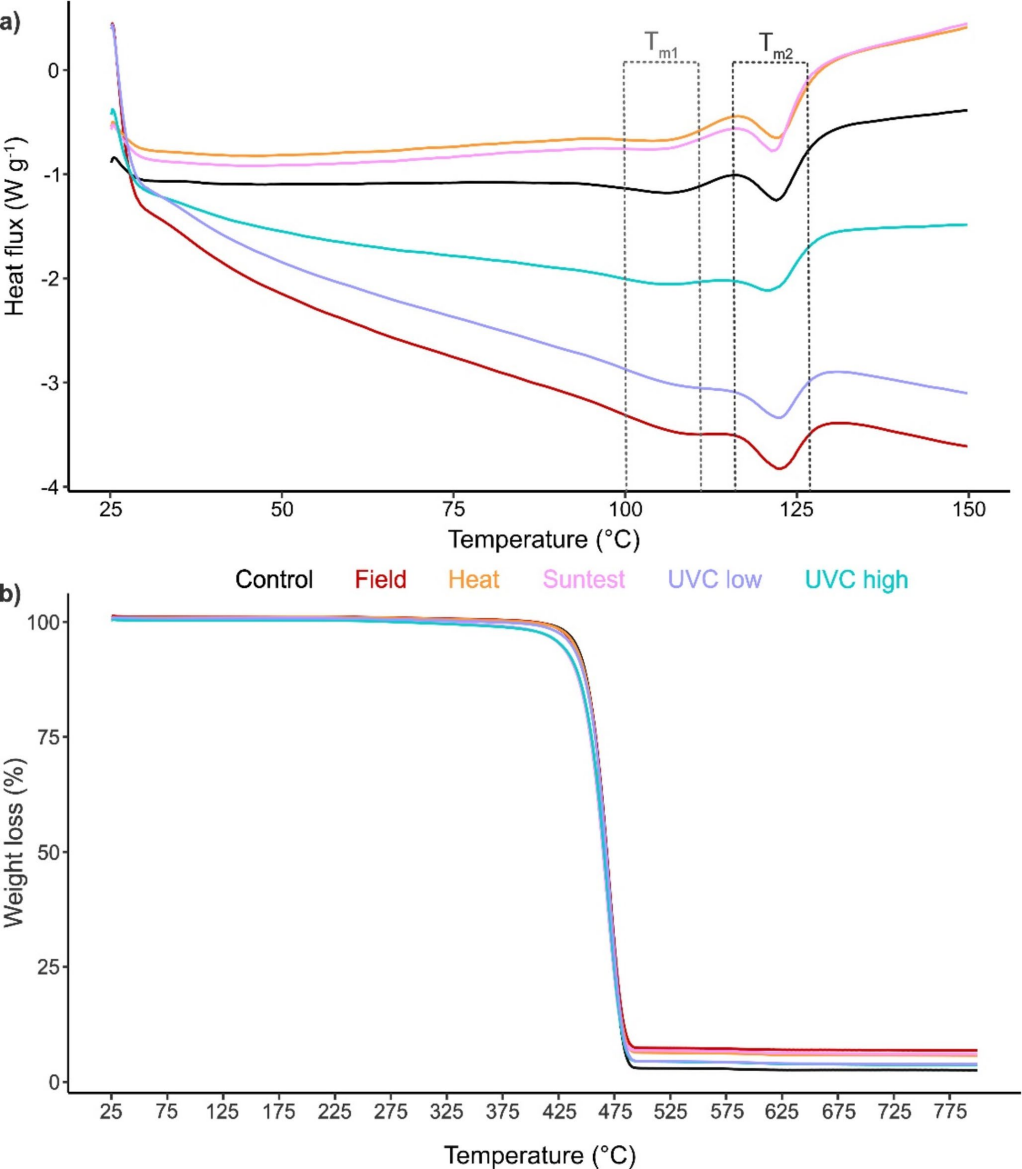
DSC **(a)** and TGA **(b)** curves of LDPE for control, field exposed samples at 6 months, and heat, Suntest (UVA), low UVC, and high UVC exposure at 20 weeks. Values expressed as mean (*n* = 3). Melting temperatures *T*_*m1*_ and *T*_*m2*_ are indicated with dotted lines

For LDPE, the DSC curves showed two separate *T*_*m*_ peaks (Fig. 5a), which is likely due to the heterogenous crystalline structure, and a slight increase of *T*_*m*_ after degradation for field and low UVC exposure, whereas heat and Suntest showed a slight reduction, with high UVC exposure remaining similar to the control (Table 1). An increase in *T*_*m*_ indicates that crosslinking has occurred during the degradation process, making the polymer more thermally stable with a higher crystallinity.

The TGA curves of degraded samples remained similar to the control, and *m*_*800*_ was low (< 7%) and increased after degradation compared to the control, with a slight increase for both UVC exposure methods and a larger increase for field, heat, and Suntest exposure methods (Fig. 5b; Table 1). This is likely due to crosslinking during the degradation process and the formation of stable degradation by-products, which are more thermally stable and increased the weight residue. There was only one *T*_*d*_, which showed little variation between samples and decreased only slightly after degradation (Fig. 5b; Table 1). A decrease in *T*_*d*_ indicates that the polymer became less thermally stable after degradation, therefore displayed a slightly earlier onset of thermal decomposition.

### Physical Polymer Changes

#### PLA/PBAT

*Ra* increased by 1.52 μm from control to 6 months field exposure (*p* < 0.001) and apart from heat samples with an increase of only 0.87 μm (*p* = 0.07) (Table 2), the other artificial degradation methods showed significant differences to the control (Table S4). Suntest samples increased by 1.81 μm (*p* = 0.003), low UVC samples increased by 2.98 μm (*p* < 0.001), and high UVC samples increased by 6.96 μm (*p* < 0.001) (Table 2, S4). *Ra* of field exposed samples was similar to heat and Suntest 20 weeks, but significantly different from both low and high UVC 20 week samples (*p* < 0.001 for all) (Table S4).

**Table 2:** Surface roughness (*Ra*) [µm] for control and selected field and treatment time exposure points

Exposure type	Ra [µm]	
	*LDPE*	*PLA/PBAT*
Control	0.81 ± 0.02	1.53 ± 0.04
Field 2 months	1.39 ± 0.06 ^NS^	1.96 ± 0.06 ^NS^
Field 4 months	1.15 ± 0.04 ^NS^	2.09 ± 0.06 ^NS^
Field 6 months	1.73 ± 0.14 ^NS^	3.05 ± 0.17 ^****^
Heat 20 weeks	7.74 ± 0.74 ^****^	2.40 ± 0.19 ^NS^
Suntest 20 weeks	1.04 ± 0.13 ^NS^	3.34 ± 0.28 ^*^
UVC low 20 weeks	1.24 ± 0.10 ^NS^	4.51 ± 0.25 ^****^
UVC high 20 weeks	1.93 ± 0.11 ^*^	8.49 ± 0.48 ^****^

Values expressed as mean ± SE. Difference to control values determined by one-way ANOVA test and level of significance expressed in superscript as follows: NS *p* > 0.05; * *p* ≤ 0.05; ** *p* ≤ 0.01; *** *p* ≤ 0.001; **** *p* ≤ 0.0001

The film thickness for PLA/PBAT mulch films increased by 2.7 μm after 6 months field exposure (*p* < 0.001) (Table 3, S4), though none of the artificial exposure methods showed significant differences to the control (Table S4). Heat and Suntest exposure increased thickness by 1.05 μm and 1.15 μm, respectively, whilst low and high UVC decreased thickness by 1 μm and 0.9 μm, respectively (Table 3). All field exposure time points were significantly different to heat and high UVC exposure (*p* < 0.001 for all), whilst only 2 and 6 months differed significantly for low UVC exposure samples (*p* < 0.001; *p* = 0.006, respectively), and all field samples were similar to Suntest (Table S4).

**Table 3:** Film thickness [µm] for control and selected field and treatment time exposure points

Exposure type	Thickness [µm]	
	*LDPE*	*PLA/PBAT*
Control	23.0 ± 0.3	10.5 ± 0.21
Field 2 months	21.2 ± 0.5 ^NS^	13.5 ± 0.39 ^****^
Field 4 months	20.6 ± 0.3 ^****^	11.7 ± 0.27 ^NS^
Field 6 months	20.2 ± 0.3 ^****^	13.2 ± 0.4 ^****^
Heat 20 weeks	24.9 ± 0.3 ^**^	9.4 ± 0.28 ^NS^
Suntest 20 weeks	23.6 ± 1.0 ^NS^	11.6 ± 0.51 ^NS^
UVC low 20 weeks	24.0 ± 0.3 ^NS^	11.5 ± 0.32 ^NS^
UVC high 20 weeks	23.1 ± 0.2 ^NS^	9.6 ± 0.21 ^NS^

Values expressed as mean ± SE. Difference to control values determined by one-way ANOVA test and level of significance expressed in superscript as follows: NS *p* > 0.05; * *p* ≤ 0.05; ** *p* ≤ 0.01; *** *p* ≤ 0.001; **** *p* ≤ 0.0001

#### LDPE

For LDPE, *Ra* increased by 0.92 μm after 6 months field exposure though no field exposure timepoints showed a significant difference to the control, similar to Suntest and low UVC samples, which increased by 0.23 μm and 0.43 μm, respectively (Table 2, S5). Whereas high UVC samples increased by 1.12 μm (*p* = 0.054), and heat samples by 6.93 μm (*p* < 0.001) (Table 2, S5). Like the control, field exposed samples were significantly different to heat samples (*p* < 0.001 for all), with no differences to other exposure methods (Table S5).

The film thickness reduced by 2.8 μm after 6 months field exposure (*p* = 0.001) (Table 3, S5). Heat showed a significant difference to the control with an increased thickness of 1.9 μm (*p* < 0.01), whilst other artificial degradation methods showed no significant differences (Table 3, S5). Suntest increased thickness by 0.6 μm, low UVC by 0.95 μm, and high UVC by 0.5 μm (Table 3). All field exposure time points were significantly different to heat exposure (*p* < 0.001 for all) and low UVC exposure (*p* < 0.01 for all) (Table S5). Suntest samples were similar to early field exposure time points but differed significantly from 3 months exposure onwards (*p* < 0.01 for all) (Table S5). High UVC exposure samples were only similar to 2 months field exposure (*p* = 0.37) but differed significantly from all other time points (*p* < 0.01 for all) (Table S5).

## Evaluation of Artificial Degradation Methods

### Reasoning Behind the Method Selection

This study focused on a subset of abiotic artificial degradation methods, due to their relevance in the initial stages of degradation of agricultural plastic mulch films, which is mainly driven by UV, temperature, moisture, and external mechanical stress [54]. The four methods were selected due to their common use in previous studies [21, 33, 55, 56], and were combined to present a comprehensive method comparison. Heat exposure was selected due to the ease of application and potential for large sample volume turnover at very low cost. Suntest exposure with irradiance in the UVA range was used as the closest comparison to natural UV exposure in a field setting, whilst UVC exposure at two intensities was selected due to its potential of accelerated degradation in a short time frame. Additionally, the accessibility and cost-effectiveness played a role in the method selection, as all methods described in this study have the potential to be carried out with minimal resources and only require access to an oven or UV lamps set up in a closed chamber [30, 33, 34].

### Efficiency of Artificial Degradation Methods

To evaluate the degradation efficiency of the four methods in terms of chemical and physical degradation of both polymers, focus was set on the overall changes to the spectra, thermal properties, surface roughness, and film thickness after 20 weeks of exposure in comparison to the control values.

For both plastic types, heat exposure was least efficient in altering functional groups of the polymers and the spectra showed only minimal change after 20 weeks of exposure, generally remaining similar to the control. It did however increase *m*_*800*_ 2.2 times for LDPE, indicating a potential formation of stable degradation by-products and the occurrence of crosslinking, which is not detected using FTIR. Whilst heat exposure caused no significant physical differences to the PLA/PBAT control, it increased the LDPE film’s thickness, and increased surface roughness 3–7 times more than the other methods.

Suntest was efficient with prolonged exposure for both mulch film types by gradually changing the abundance of functional groups. Both polymers showed no indicative directional distribution in the NMDS plots, but the overall spectra changes followed a linear trend with increasing exposure time. This method showed a high variability in CH and C=O peak area changes for individual time points, but generally differed from the control at later exposure times. For both polymers, *m*_*800*_ increased whereas *T*_*d1−3*_ and *T*_*m1−2*_, decreased, indicating a production of thermally stable by-products but overall reduction in thermal stability of the polymer. In contrast to heat exposure, Suntest caused no significant physical changes to LDPE but slightly increased the surface roughness of PLA/PBAT films.

In contrast to the previous two methods, the efficiency of low UVC exposure differed between mulch film types. Whilst the spectra showed a clear decrease in peak intensities and reduction of the C=O and CH peak areas for PLA/PBAT, they remained similar to the control for LDPE after 20 weeks. This is reflected in only slight changes to *m*_*800*_ and *T*_*m1*_ for LDPE, whereas PLA/PBAT showed a higher *m*_*800*_ increase and a large decrease of *T*_*d1*_ with minor decreases of *T*_*d2−3*_, indicating a reduction in thermal stability. As with Suntest exposure, physical properties of LDPE remained close to the control values with surface roughness increasing in PLA/PBAT.

When looking at the spectra changes for both polymers, high UVC was extremely efficient in reducing C=O and CH peak areas for PLA/PBAT and increasing the C=O area for LDPE after 20 weeks exposure, as well as increasing surface roughness but not thickness of the plastic mulch films. High UVC reduced the thermal stability of both polymers, though more pronounced for PLA/PBAT where *T*_*d2*_ was no longer identifiable.

In summary, high UVC exposure was the most efficient artificial degradation method to change peak intensities in both polymer spectra and alter their thermal stability, followed by low UVC, Suntest, and lastly heat. Heat had the largest effect on LDPE’s film thickness and surface roughness, whereas PLA/PBAT was most affected by Suntest and both UVC exposure intensities for surface roughness changes.

### Degradation Rate of Artificial Degradation Methods

By evaluating the degradation rate of the different methods, an assessment of minimum required exposure time until changes to the control become apparent for each method can be made. This assessment focusses on the intermediate time points of 5, 10, 15, and 20 weeks and the ongoing changes in spectra compared to the control films.

Whilst a short-term heat exposure was sufficient for changing peak areas for PLA/PBAT, no changes were observed for LDPE. The clustering of exposure time points, though more pronounced for LDPE, indicates that spectra were changing but not in a linear fashion with increasing exposure time. Whilst the C=O and CH peak areas of LDPE remained similar to the control throughout the 20 weeks, they differed significantly from the control for the early time points for PLA/PBAT, with no difference for later times. Overall, this indicates that changes to LDPE through heat exposure alone would likely take significantly more time than 20 weeks, but can occur [21], whilst short-term exposure can cause short-term changes to PLA/PBAT, though spectra seemingly become more similar to the control again with prolonged exposure time.

Suntest exposure ranked third in terms of degradation rate for both polymers, only showing significant differences to the control peak areas after 15 weeks of exposure.

Low UVC exposure affected the chemical structure of the PLA/PBAT film extremely quickly, with spectra differing significantly from the control after only 5 weeks, and peak intensities steadily decreasing with prolonged exposure. Although the LDPE CH peak area differed significantly from the control after only 5 weeks of exposure, there was no change in the C=O area, indicating that a longer exposure period would be required for oxidation of LDPE.

Likewise, high UVC accelerated the reduction in peak intensity for PLA/PBAT, yielding significant differences after only 5 weeks, whereas significant increases to the C=O area of LDPE only became apparent after 15 weeks.

Overall, the degradation rate of the four methods differed between polymer types. Heat exposure was slowest for both plastic mulch films, although short-term changes could be achieved after 5–10 weeks of exposure of PLA/PBAT. Suntest exposure ranked similar for both LDPE and PLA/PBAT, with significant spectra changes after 15 weeks exposure. Whilst both low and high UVC exposure significantly reduced peak intensity in PLA/PBAT after only 5 weeks, only high UVC exposure increased the C=O peak area for LDPE after 15 weeks. The differing response time for PLA/PBAT and LDPE films likely reflects the different chemical bonds broken during UV degradation. For both PLA and PBAT in the biodegradable film, chain scission proceeds via photocleavage of ester bonds in the polymer chain, yielding vinyl and carboxylic acid end groups. The C-C backbone of LDPE is comparatively more stable, and degrades via radical formation under UV, which can initiate oxidation under oxic conditions. However, the semi-crystalline regions in LDPE can reduce oxygen penetration of the film, resulting in radical quenching to form alkyl chains rather than oxygenated degradation product (e.g. carboxylic acids, aldehydes, alkanols). In addition, antioxidants included in the LDPE film may quench radicals produced by UV action. Overall, the underlying chemical differences between the two films, and their additives, are reflected in the different degradation patterns observed via FTIR and thermal methods.

### Similarity of Artificial Methods to Natural Field Degradation of Mulch Films

To assess the similarity of the artificial degradation methods to realistic field degradation, the focus was on the comparison of 5, 10, 15, and 20 weeks exposure of the artificial methods to field exposure time points, evaluating spectra and physical changes. Thermal properties were compared on only 20 weeks artificial exposure and 6 months field exposure time points.

Heat exposure was not a suitable method for mimicking realistic field weathering for either PLA/PBAT or LDPE. This is evidenced by the contrasting peak area increase for both C=O and CH for PLA/PBAT to the decrease observed during field exposure, indicating that heat exposure changes the polymer in a very different way to natural exposure. Thermal stability of heat exposed samples is also higher than for field exposure, which might be attributed to the 20 weeks thermal exposure resulting in crosslinking or increased crystallinity. Similarly, the overall C=O peak area reduction and CH area increase contrasts the trends observed for LDPE field samples, with significant differences in surface roughness and film thickness. As this is the only method without any type of UV exposure and is also the most different to the field exposed film, this indicates the importance of UV mechanisms for polymer degradation in field conditions, which are better reflected by the other methods for artificial degradation presented herein.

In contrast, Suntest exposure proved to be a more suitable method to replicate field degradation of both mulch film types, aligning with previous findings of creating field-realistic changes in other polymer types [18, 19]. The overall spectra changes followed a similar pattern to the field samples and the physical mulch film attributes were similar to all field exposure time points, except for film thickness changes in LDPE. However, the C=O and CH peak areas were extremely variable throughout, with short artificial exposure time being more similar to shorter field exposure time and vice versa for PLA/PBAT, but showed no clear indicative trend for LDPE. The thermal decomposition onset was lower for both polymers compared to field exposed samples, accompanied by a lower *T*_*m*_ in LDPE and increased *m*_*800*_ in PLA/PBAT, indicating an overall lower thermal stability.

A short exposure time of low UVC is sufficient to simulate field degradation for both plastic types. When looking at the overall spectra change, low UVC exposure does not seem like an obvious good fit for mimicking field degradation for either polymer type. However, when focusing on the peak area changes of PLA/PBAT and their similarity to field exposure time points, the similarities seem to develop in a parallel fashion between both exposure methods. Early low UVC samples (5 weeks) only bear similarity to early field samples (2 months) but become more similar to later field samples (6 months) with increasing exposure time. This stands in contrast to the increased *m*_*800*_ and lower *T*_*d1*_ of PLA/PBAT, indicating a difference in degradation processes which affect overall thermal stability. For LDPE, low UVC samples apart from 20 weeks were similar to all field time points, which is supported by a similar observed thermal stability. However, low UVC exposure yielded a lower residual mass compared to field exposure, suggesting the occurrence of less crosslinking and production of more stable degradation by-products. The physical properties of both plastic types were differentially affected by the low UVC exposure, with a significant change in surface roughness but not so much thickness for PLA/PBAT and vice versa for LDPE.

A prolonged exposure to high UVC was not suitable for either polymer type if attempting to simulate field degradation. The high UVC irradiation reduced and smoothed the PLA/PBAT C=O peak drastically even after only 5 weeks, which contrasts the gradual change inflicted through field exposure. This is reflected in the large reduction of *T*_*d1−2*_ compared to field exposed samples, indicating a highly reduced thermal stability. In contrast, prolonged high UVC exposure increased the C=O peak area for LDPE, making the spectra more dissimilar to field exposed samples with increasing exposure time, with the most similar time points being 5 and 10 weeks. The slightly lower *T*_*m1−2*_, *T*_*d*_, and *m*_*800*_ support the assumption that high UVC exposure affects the degradation process in different ways to field exposure. The harsh effect of high UVC irradiance translated into a significant difference of surface roughness and thickness to all field exposure time points for PLA/PBAT and an increased thickness for LDPE compared to 3–6 months.

In summary, the best suited method for simulating realistic field degradation was Suntest, as the overall spectra, thermal, and physical parameters for both plastic mulch films changed in the most similar way to field exposure. This is followed by the low UVC exposure for a short period of time, although it affected the physical properties of both polymer types and prolonged exposure was not suitable. The high UVC exposure for a short period of time was suitable for LDPE, although it affected both surface roughness and thickness, but it was too intense for PLA/PBAT, due to the higher susceptibility of the polymer to UV-mediated chain scission compared to LDPE. Heat on the other hand did not change the spectra sufficiently and affected the physical properties for LDPE. A summary overview of all artificial degradation methods, evaluated factors, and criteria can be found in Table 4.

**Table 4:** Overview of artificial degradation methods of LDPE and PLA/PBAT plastic mulch film after 20 weeks exposure in comparison to field-aged plastic mulch film characteristics after 6 months

	LDPE				PLA/PBAT			
	Heat	Suntest (UVA)	UVC (low irradiance)	UVC (high irradiance)	Heat	Suntest (UVA)	UVC (low irradiance)	UVC (high irradiance)
C=O peak area	↔	↔	↔	↑	↑	↔	↔	↓
CH peak area	↑	↔	↑	↑	↑	↑	↑	↑
Carbonyl index	↓	↔	↓	↓	↓	↓	↓	↓
Residual mass (*m*_*800*_)	↑	↑	↔	↔	↔	↑	↑	↑
Onset decomposition temperature (*T*_*d*_)	↔	↓	↔	↓	↔	↓	↓	↓
Melting temperature (*T*_*m*_)	↓	↓	↑	↔	n/a	n/a	n/a	n/a
Film thickness	↑	↑	↑	↑	↓	↔	↓	↓
Surface roughness	↑	↔	↔	↔	↔	↑	↑	↑
Degradation efficiency	4	3	2	1	4	3	2	1
Degradation rate	4	1–2	3	1	4	3	1–2	1
Similarity to field exposure	4	1	2	2–3	4	1	2	3–4

Arrows indicate increase (↑), decrease (↓), or no significant difference (↔) after 20 weeks exposure. Artificial degradation methods are ranked according to their degradation efficiency, rate, and similarity to field-exposed samples (after 6 months) on a scale from 1–4, with 1 being most suitable and 4 being least suitable

It is important to note that field exposed samples were subjected to not only UV irradiance, but mechanical stress, precipitation, heat, humidity, and microbial degradation through direct contact with the soil surface, which are all factors influencing the degradation mechanisms and dynamics of the two respective mulch films. This complex interaction of factors driving degradation explains why there is no single artificial degradation method to completely replicate natural weathering, and that depending on geographical location, time of year, and environmental variables, field degraded materials will differ from each other as well. We therefore recommend any follow-up studies on artificial degradation comparisons to include a combination of methods used here, as well as any other environmentally relevant factors (e.g. mechanical stress, humidity) to build on the results of this study.

## Conclusion

Here, we compared four abiotic artificial degradation methods and evaluated their efficiency, rate, and similarity to realistic field degradation using one conventional and one biodegradable plastic mulch film. We determined that the overall efficiency for spectra changes of the methods ranked high UVC > low UVC > Suntest > heat, also reflecting the respective strengths of the degradation methods, with both UVC exposure methods significantly changing the physical film properties of PLA/PBAT, and heat most affecting LDPE. We attribute this order to the decrease in exposure intensity from high UVC exposure to only heat. In terms of degradation rate, we found that PLA/PBAT can be degraded fastest at both UVC intensities, followed by Suntest and heat, whereas LDPE showed a similar degradation rate for Suntest and high UVC exposure. The methods yielding most similar spectra changes compared to realistic field degradation for PLA/PBAT were Suntest, followed by short periods of low UVC exposure, with high UVC and heat exposure being unsuitable, likely as they are the most and least intense methods. Methods for LDPE ranked similarly, with Suntest followed by short periods of low or high UVC exposure, and heat not being suitable to recreate realistic degradation.

In conclusion, we recommend Suntest exposure as the most suitable and realistic artificial degradation method at a medium rate, UVC exposure if the aim is rapid degradation without the need to simulate realistic changes, and heat for artificially degrading large volumes of samples if there is no requirement for realistic degradation and sufficient time is available.

As methods in this study yielded differential results depending on what parameter was examined, we advise researchers to first establish the overall degradation aim, i.e., changes in chemical, thermal, physical, or mechanical properties, to select the most appropriate artificial degradation method. For chemical degradation, we also advise to specify the area of interest within the spectra prior to method selection, i.e., focus on overall spectra changes or only particular functional groups. Lastly, we have shown that the methods tested in this study yield different results depending on polymer type, we therefore strongly recommend taking this into account during method selection.

For future work, we recommend studies to assess the degradation of artificially degraded materials against naturally degraded reference materials, to place artificially degraded materials into an environmentally relevant context. We also recommend quantifying the achieved artificial degradation against non-degraded materials or realistic reference materials, to provide comparability across studies. Although we appreciate that creating realistic reference materials is not always feasible due to time and resource constraints, sourcing data from published studies that have used similar materials in realistic settings would be useful.

## Electronic Supplementary Material

Below is the link to the electronic supplementary material.

Supplementary Material 1

## Data Availability

All data will be made available upon request.
