# Supplementary material for: Assessment of Four Artificial Methods for Aging Plastic Mulch Films According to Efficiency, Rate, and Similarity to Natural Field-Aged Plastics
Source: J Polym Environ. 2025 Jan 18;33(3):1542–60. doi: 10.1007/s10924-024-03481-5 (PMC11829909; doi:10.1007/s10924-024-03481-5)
Supplement: Supplementary file 1 — Supplementary Material 1 [file 10924_2024_3481_MOESM1_ESM.docx]

Supplementary information

**Assessment of four artificial methods for aging plastic mulch films according to efficiency, rate, and similarity to natural field-aged plastics**

Martine Graf ^1*^, Michaela K. Reay ^2^, Athanasios Dimitriou ^3^, David R. Chadwick ^1^, & Davey L. Jones ^1^

*^1^ School of Environmental & Natural Sciences, Bangor University, Bangor, LL57 2 UW, UK*

*^2^ Organic Geochemistry Unit, School of Chemistry, University of Bristol, Bristol, BS8 1TS, UK*

*^3^ The BioComposites Centre, Bangor University, Bangor, LL57 2UW, UK*

Corresponding author: * Martine Graf

Corresponding author address: School of Environmental & Natural Sciences, Bangor University, Bangor, Gwynedd, LL57 2UW, UK

Corresponding author e-mail: m.graf@bangor.ac.uk

ORCID: 0000-0001-7994-0529

**
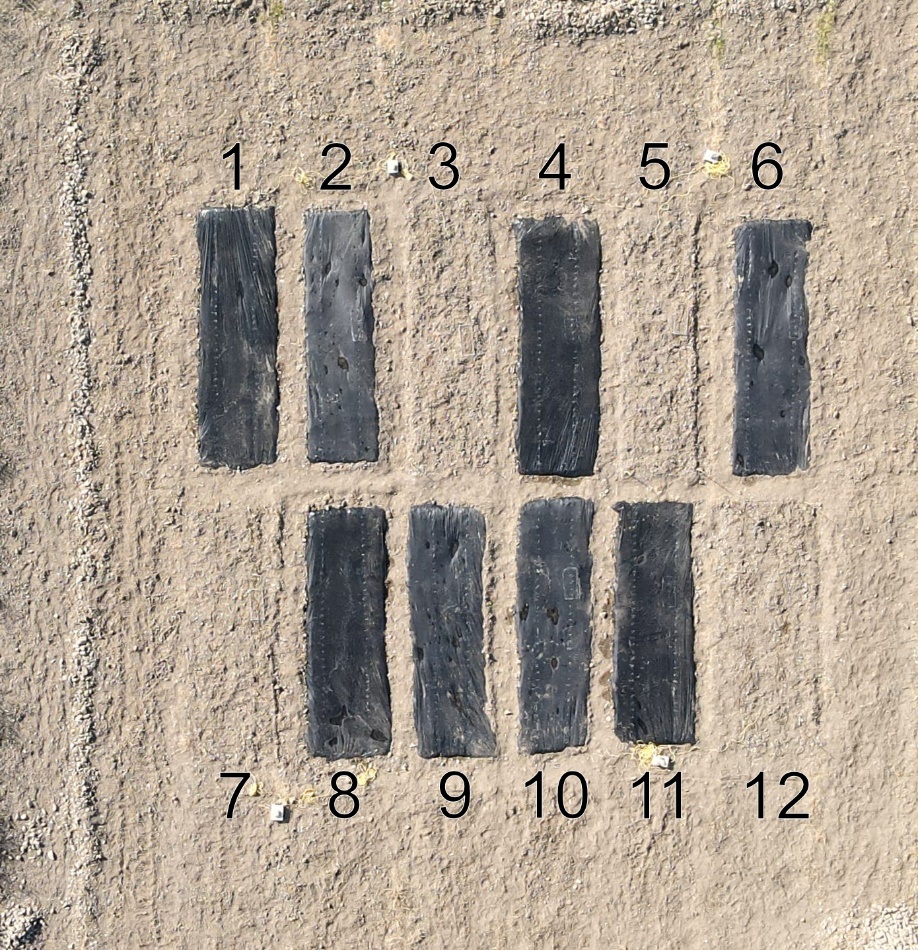
**

**Fig. S1** Experimental field set-up for natural weathering of LDPE and PLA/PBAT mulch films under maize cropping at the start of the growing season. Plots 1, 4, 8, 11 are covered with LDPE mulch film; plots 2, 6, 9, 10 are covered with PLA/PBAT mulch film; plots 3, 5, 7, 12 have no mulch film coverage

**
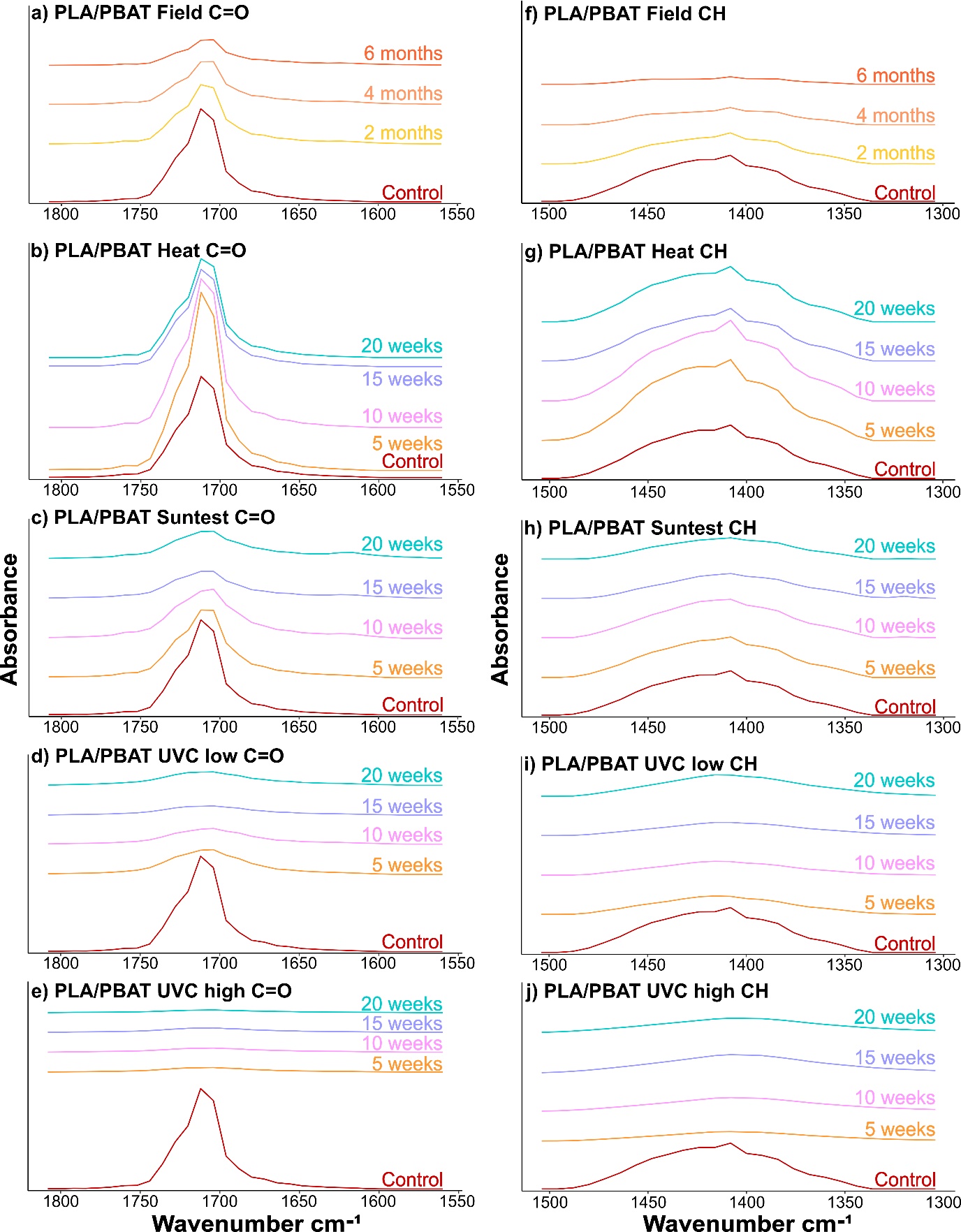
**

**Fig. S2** ATR-FTIR absorbance spectra for C=O and CH regions of interest for PLA/PBAT. Spectra are expressed as mean (*n* = 20). Graphs show a selection of time points for **a)** Field exposure C=O region; **b)** Heat exposure C=O region; **c)** Suntest (UVA) exposure C=O region; **d)** UVC exposure at low intensity C=O region; **e)** UVC exposure at high intensity C=O region; **f)** Field exposure CH region; **g)** Heat exposure CH region; **h)** Suntest (UVA) exposure CH region; **i)** UVC exposure at low intensity CH region; **j)** UVC exposure at high intensity CH region

**
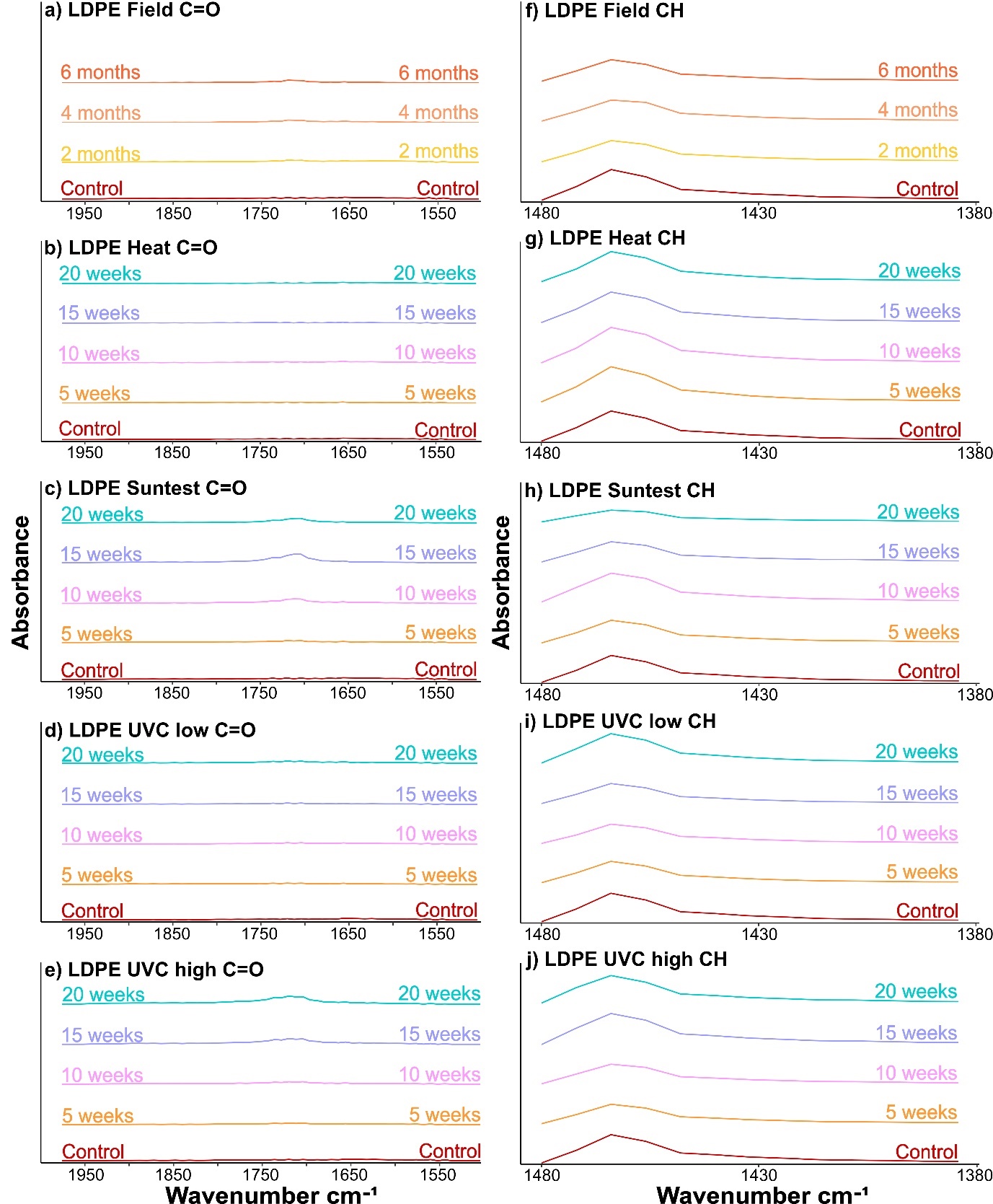
**

**Fig. S3** ATR-FTIR absorbance spectra for C=O and CH regions of interest for LDPE. Spectra are expressed as mean (*n* = 20). Graphs show a selection of time points for **a)** Field exposure C=O region; **b)** Heat exposure C=O region; **c)** Suntest (UVA) exposure C=O region; **d)** UVC exposure at low intensity C=O region; **e)** UVC exposure at high intensity C=O region; **f)** Field exposure CH region; **g)** Heat exposure CH region; **h)** Suntest (UVA) exposure CH region; **i)** UVC exposure at low intensity CH region; **j)** UVC exposure at high intensity CH region

**Table S1** Environmental conditions for field exposure of LDPE and PLA/PBAT plastic mulch film for the duration of one growing season (6 months) in 2022.

| **Month** | **Exposure time** | **Average monthly air temperature [°C]** | **Total monthly precipitation [mm]** | **Average monthly relative humidity [%]** | **Average solar radiation [W m^-2^]** | **Average wind speed**  **[km h^-1^]** |
| --- | --- | --- | --- | --- | --- | --- |
| June | 1 month | 14.9 | 41 | 76.7 | 222.3 | 10.1 |
| July | 2 months | 17.5 | 33 | 76.6 | 183.2 | 8.04 |
| August | 3 months | 17.7 | 64 | 79.3 | 189.3 | 7.68 |
| September | 4 months | 15.0 | 85 | 81.0 | 95.0 | 7.89 |
| October | 5 months | 14.0 | 125 | 79.3 | 66.3 | 14.5 |
| November | 6 months | 11.2 | 154 | 78.0 | 28.8 | 16.2 |
| Average/Cumulative^†^ | | 15.2 | 502^†^ | 78.5 | 132.7 | 10.7 |

**Table S2** PLA/PBAT C=O (1560 – 1808 cm^-1^) and CH (1304 – 1504 cm^-1^) peak area differences (*n* = 20). *p* values determined by One-way ANOVA with subsequent Tukey HSD test (*df* = 19, *F* = 67.06 & 53.74 for C=O and CH, respectively). Significant differences between treatments in bold (*p* ≤ 0.05).

|  | ***C=O peak area difference p values*** | | | | ***CH peak area difference p values*** | | | |
| --- | --- | --- | --- | --- | --- | --- | --- | --- |
|  | *Control* | *Field 2*  *months* | *Field 4*  *months* | *Field 6*  *months* | *Control* | *Field 2*  *months* | *Field 4*  *months* | *Field 6*  *months* |
| *Field 2 months* | 0.06 |  |  |  | **<0.001** |  |  |  |
| *Field 4 months* | **<0.001** | 0.89 |  |  | **<0.001** | **<0.01** |  |  |
| *Field 6 months* | **<0.001** | **<0.001** | 0.54 |  | **<0.001** | **<0.001** | 0.38 |  |
| *Heat 5 weeks* | **<0.001** | **<0.001** | **<0.001** | **<0.001** | **<0.001** | **<0.001** | **<0.001** | **<0.001** |
| *Heat 10 weeks* | **<0.001** | **<0.001** | **<0.001** | **<0.001** | **<0.001** | **<0.001** | **<0.001** | **<0.001** |
| *Heat 15 weeks* | 0.99 | 0.36 | **<0.001** | **<0.001** | 0.99 | **0.01** | **<0.001** | **<0.001** |
| *Heat 20 weeks* | 0.99 | 0.16 | **<0.001** | **<0.001** | 0.99 | **<0.001** | **<0.001** | **<0.001** |
| *Suntest 5 weeks* | 0.99 | 0.61 | **0.001** | **<0.001** | 0.99 | **0.05** | **<0.001** | **<0.001** |
| *Suntest 10 weeks* | 0.91 | 0.99 | 0.054 | **<0.001** | 0.98 | 0.21 | **<0.001** | **<0.001** |
| *Suntest 15 weeks* | **<0.001** | 0.13 | 0.99 | 0.99 | **<0.001** | 0.69 | 0.97 | **<0.01** |
| *Suntest 20 weeks* | **<0.001** | 0.86 | 0.99 | 0.58 | **<0.001** | 0.99 | 0.33 | **<0.001** |
| *UVC low 5 weeks* | **<0.001** | 0.07 | 0.99 | 0.99 | **<0.001** | 0.25 | 0.99 | **0.02** |
| *UVC low 10 weeks* | **<0.001** | **<0.001** | 0.89 | 0.99 | **<0.001** | **<0.01** | 0.99 | 0.68 |
| *UVC low 15 weeks* | **<0.001** | **<0.001** | **<0.001** | 0.82 | **<0.001** | **<0.01** | 0.99 | 0.52 |
| *UVC low 20 weeks* | **<0.001** | **<0.001** | 0.07 | 0.99 | **<0.001** | 0.99 | 0.48 | **<0.001** |
| *UVC high 5 weeks* | **<0.001** | **<0.001** | **<0.001** | **0.047** | **<0.001** | **<0.001** | 0.99 | 0.99 |
| *UVC high 10 weeks* | **<0.001** | **<0.001** | **<0.001** | **0.04** | **<0.001** | **0.02** | 0.99 | 0.19 |
| *UVC high 15 weeks* | **<0.001** | **<0.001** | **<0.001** | **0.05** | **<0.001** | 0.91 | 0.85 | **<0.001** |
| *UVC high 20 weeks* | **<0.001** | **<0.001** | **<0.001** | **<0.01** | **<0.001** | 0.21 | 0.99 | **0.02** |

**Table S3** LDPE C=O (1504 – 1976 cm^-1^) and CH (1384 – 1480 cm^-1^) peak area differences (*n* = 20). *p* values determined by One-way ANOVA with subsequent Tukey HSD test (*df* = 19; *F* = 28.55 & 21.73 for C=O and CH, respectively). Significant differences between treatments in bold (*p* ≤ 0.05).

|  | ***C=O peak area difference p values*** | | | | ***CH peak area difference p values*** | | | |
| --- | --- | --- | --- | --- | --- | --- | --- | --- |
|  | *Control* | *Field 2 months* | *Field 4 months* | *Field 6 months* | *Control* | *Field 2 months* | *Field 4 months* | *Field 6 months* |
| *Field 2 months* | 0.99 |  |  |  | **<0.001** |  |  |  |
| *Field 4 months* | 0.99 | 0.99 |  |  | **<0.001** | 0.99 |  |  |
| *Field 6 months* | 0.99 | 0.99 | 0.99 |  | **<0.001** | 0.99 | 0.99 |  |
| *Heat 5 weeks* | 0.96 | 0.37 | 0.19 | 0.32 | 0.99 | **<0.001** | **<0.001** | **<0.001** |
| *Heat 10 weeks* | 0.99 | 0.99 | 0.98 | 0.99 | 0.85 | **<0.001** | **<0.001** | **<0.001** |
| *Heat 15 weeks* | 0.8 | 0.15 | 0.06 | 0.11 | 0.99 | **<0.01** | **<0.01** | **<0.001** |
| *Heat 20 weeks* | 0.88 | 0.21 | 0.09 | 0.17 | 0.99 | **0.01** | **<0.01** | **<0.001** |
| *Suntest 5 weeks* | 0.99 | 0.98 | 0.92 | 0.97 | 0.34 | 0.77 | 0.71 | 0.32 |
| *Suntest 10 weeks* | 0.06 | 0.59 | 0.81 | 0.66 | 0.99 | **<0.001** | **<0.001** | **<0.001** |
| *Suntest 15 weeks* | **<0.001** | **<0.001** | **<0.001** | **<0.001** | **<0.01** | 0.99 | 0.99 | 0.99 |
| *Suntest 20 weeks* | 0.96 | 0.99 | 0.99 | 0.99 | **<0.001** | **<0.01** | **<0.01** | 0.06 |
| *UVC low 5 weeks* | 0.98 | 0.49 | 0.32 | 0.44 | **<0.001** | 0.99 | 0.99 | 0.99 |
| *UVC low 10 weeks* | 0.99 | 0.99 | 0.96 | 0.99 | **<0.001** | 0.99 | 0.99 | 0.99 |
| *UVC low 15 weeks* | 0.98 | 0.51 | 0.29 | 0.45 | **<0.001** | 0.99 | 0.99 | 0.99 |
| *UVC low 20 weeks* | 0.99 | 0.99 | 0.99 | 0.99 | 0.99 | **<0.001** | **<0.001** | **<0.001** |
| *UVC high 5 weeks* | 0.99 | 0.93 | 0.78 | 0.9 | **<0.001** | 0.99 | 0.99 | 0.99 |
| *UVC high 10 weeks* | 0.99 | 0.99 | 0.99 | 0.99 | **<0.001** | 0.99 | 0.99 | 0.99 |
| *UVC high 15 weeks* | **<0.001** | **<0.001** | **<0.001** | **<0.001** | 0.69 | **<0.001** | **<0.001** | **<0.001** |
| *UVC high 20 weeks* | **<0.001** | **<0.001** | **<0.001** | **<0.001** | 0.99 | **<0.001** | **<0.001** | **<0.001** |

**Table S4** Differences between PLA/PBAT control, and selected field and treatment exposure time points for film thickness (µm) and surface roughness (*Ra*) [µm] (*n* = 20). *p* values determined by One-way ANOVA with subsequent Tukey HSD test (*df* = 10; *F* = 19.3 & 99.82 for thickness and surface roughness respectively). Significant differences between treatments in bold (*p* ≤ 0.05).

|  |  | **Thickness – *p* values for treatment differences after One-way ANOVA and Tukey HSD test** | | | | | | | | | | |
| --- | --- | --- | --- | --- | --- | --- | --- | --- | --- | --- | --- | --- |
|  |  | *Control* | *Field 1 month* | *Field 2 months* | *Field 3 months* | *Field 4 months* | *Field 5 months* | *Field 6 months* | *Heat 20 weeks* | *Suntest 20 weeks* | *UVC low 20 weeks* | *UVC high 20 weeks* |
| ***Ra* – *p* values for treatment differences after One-way ANOVA and Tukey HSD test** | *Control* |  | **<0.01** | **<0.001** | **<0.001** | 0.14 | **0.013** | **<0.001** | 0.37 | 0.85 | 0.44 | 0.60 |
|  | *Field 1 month* | 0.59 |  | 0.18 | 0.99 | 0.95 | 0.99 | 0.68 | **<0.001** | 0.99 | 0.68 | **<0.001** |
|  | *Field 2 months* | 0.89 | 0.99 |  | 0.76 | **0.003** | **0.04** | 0.99 | **<0.001** | 0.18 | **<0.001** | **<0.001** |
|  | *Field 3 months* | 0.38 | 0.99 | 0.99 |  | 0.44 | 0.92 | 0.99 | **<0.001** | 0.88 | 0.14 | **<0.001** |
|  | *Field 4 months* | 0.63 | 0.99 | 0.99 | 0.99 |  | 0.99 | **0.04** | **<0.001** | 0.99 | 0.99 | **<0.001** |
|  | *Field 5 months* | **0.013** | 0.88 | 0.56 | 0.97 | 0.86 |  | 0.29 | **<0.001** | 0.99 | 0.95 | **<0.001** |
|  | *Field 6 months* | **<0.001** | **0.03** | **<0.01** | 0.08 | **0.03** | 0.77 |  | **<0.001** | 0.47 | **0.006** | **<0.001** |
|  | *Heat 20 weeks* | 0.07 | 0.99 | 0.88 | 0.99 | 0.98 | 0.99 | 0.42 |  | 0.06 | **<0.001** | 0.99 |
|  | *Suntest 20 weeks* | **<0.01** | 0.16 | 0.07 | 0.25 | 0.15 | 0.77 | 0.99 | 0.55 |  | 0.99 | 0.11 |
|  | *UVC low 20 weeks* | **<0.001** | **<0.001** | **<0.001** | **<0.001** | **<0.001** | **<0.001** | **<0.001** | **<0.001** | 0.22 |  | **<0.01** |
|  | *UVC high 20 weeks* | **<0.001** | **<0.001** | **<0.001** | **<0.001** | **<0.001** | **<0.001** | **<0.001** | **<0.001** | **<0.001** | **<0.001** |  |

**Table S5** Differences between LDPE control, and selected field and treatment exposure time points for film thickness (µm) and surface roughness (*Ra*) [µm] (*n* = 20). *p* values determined by One-way ANOVA with subsequent Tukey HSD test (*df* = 10; *F* = 15.38 & 62.45 for thickness and surface roughness respectively). Significant differences between treatments in bold (*p* ≤ 0.05).

|  |  | **Thickness – *p* values for treatment differences after One-way ANOVA and Tukey HSD test** | | | | | | | | | | |
| --- | --- | --- | --- | --- | --- | --- | --- | --- | --- | --- | --- | --- |
|  |  | *Control* | *Field 1 month* | *Field 2 months* | *Field 3 months* | *Field 4 months* | *Field 5 months* | *Field 6 months* | *Heat 20 weeks* | *Suntest 20 weeks* | *UVC low 20 weeks* | *UVC high 20 weeks* |
| ***Ra* – *p* values for treatment differences after One-way ANOVA and Tukey HSD test** | *Control* |  | **<0.01** | 0.45 | **<0.001** | **<0.001** | **<0.001** | **<0.001** | **<0.01** | 0.99 | 0.60 | 0.99 |
|  | *Field 1 month* | 0.76 |  | 0.91 | **0.03** | 0.94 | **0.02** | 0.45 | **<0.001** | 0.053 | **<0.001** | **<0.01** |
|  | *Field 2 months* | 0.83 | 0.99 |  | **<0.001** | 0.12 | **<0.001** | **<0.01** | **<0.001** | 0.45 | **<0.01** | 0.37 |
|  | *Field 3 months* | 0.98 | 0.99 | 0.99 |  | 0.67 | 0.99 | 0.99 | **<0.001** | **<0.001** | **<0.001** | **<0.001** |
|  | *Field 4 months* | 0.99 | 0.99 | 0.99 | 0.99 |  | 0.52 | 0.99 | **<0.001** | **<0.01** | **<0.001** | **<0.001** |
|  | *Field 5 months* | 0.15 | 0.99 | 0.98 | 0.87 | 0.74 |  | 0.97 | **<0.001** | **<0.001** | **<0.001** | **<0.001** |
|  | *Field 6 months* | 0.22 | 0.99 | 0.99 | 0.93 | 0.84 | 0.99 |  | **<0.001** | **<0.001** | **<0.001** | **<0.001** |
|  | *Heat 20 weeks* | **<0.001** | **<0.001** | **<0.001** | **<0.001** | **<0.001** | **<0.001** | **<0.001** |  | 0.78 | 0.60 | **<0.01** |
|  | *Suntest 20 weeks* | 0.99 | 0.99 | 0.99 | 0.99 | 0.99 | 0.95 | 0.97 | **<0.001** |  | 0.99 | 0.99 |
|  | *UVC low 20 weeks* | 0.98 | 0.99 | 0.99 | 0.99 | 0.99 | 0.88 | 0.94 | **<0.001** | 0.99 |  | 0.67 |
|  | *UVC high 20 weeks* | 0.054 | 0.94 | 0.91 | 0.64 | 0.47 | 0.99 | 0.99 | **<0.001** | 0.87 | 0.66 |  |
